# Machine learning identifies clinical sepsis phenotypes that translate to the plasma proteome

**DOI:** 10.1007/s15010-025-02628-3

**Published:** 2025-08-28

**Authors:** Thilo Bracht, Maike Weber, Kerstin Kappler, Lars Palmowski, Malte Bayer, Karin Schork, Tim Rahmel, Matthias Unterberg, Helge Haberl, Alexander Wolf, Björn Koos, Katharina Rump, Dominik Ziehe, Ulrich Limper, Dietrich Henzler, Stefan Felix Ehrentraut, Thilo von Groote, Alexander Zarbock, Katrin Marcus-Alic, Martin Eisenacher, Michael Adamzik, Barbara Sitek, Hartmuth Nowak

**Affiliations:** 1 Department of Anesthesiology, Intensive Care Medicine and Pain Therapy, University Hospital Knappschaftskrankenhaus Bochum, Bochum, Germany; 2 Medizinisches Proteom-Center, Ruhr-University Bochum, Bochum, Germany; 3 Center for Protein Diagnostics (PRODI), Medical Proteome Analysis, Ruhr-University Bochum, Bochum, Germany; 4 Core Unit Bioinformatics, Medical Faculty, CUBiMed.RUB, Ruhr University Bochum, Bochum, Germany; 5 Center for Artificial Intelligence, Medical Informatics and Data Science, University Hospital Knappschaftskrankenhaus Bochum, Bochum, Germany; 6 Department of Anesthesiology and Operative Intensive Care Medicine, University of Witten/Herdecke, Cologne Merheim Medical School, 51109 Cologne, Germany; 7 Department of Anesthesiology, Surgical Intensive Care, Emergency and Pain Medicine, Ruhr-University Bochum, Klinikum Herford, 32049 Herford, Germany; 8 Klinik für Anästhesiologie und Operative Intensivmedizin, Universitätsklinikum Bonn, 53127 Bonn, Germany; 9 Department of Anesthesiology, Intensive Care and Pain Medicine, University Hospital of Münster, Münster, Germany

**Keywords:** Artificial intelligence, Proteomics, Sepsis, Cluster analysis, Precision medicine, Mass spectrometry

## Abstract

**Background:**

Sepsis therapy is still limited to treatment of the underlying infection and supportive measures. To date, various sepsis subtypes were proposed, but therapeutic options addressing the molecular changes of sepsis were not identified. With the aim of a future individualized therapy, we used machine learning (ML) to identify clinical phenotypes and their temporal development in a prospective, multicenter sepsis cohort and characterized them using plasma proteomics.

**Methods:**

Routine clinical data and blood samples were collected from 384 patients. Sepsis phenotypes were identified based on clinical measurements and plasma samples from 301 patients were analyzed using mass spectrometry. The obtained data were evaluated in relation to the phenotypes, and supervised ML models were developed enabling prospective phenotype classification.

**Results:**

Three sepsis phenotypes were identified. Cluster C was characterized by the highest disease severity and multi-organ failure with leading liver failure. Cluster B showed relevant organ failure, with renal damage being particularly prominent in comparison to cluster A. Time course analysis showed a strong association of cluster C with mortality, while patients in cluster B were likely to change the cluster until day 4. The plasma proteome reflected the clinical features of the phenotypes and showed gradual consumption of complement and coagulation factors with increasing sepsis severity. Supervised ML models allowed the assignment of patients based on only seven widely available features (alanine transaminase (ALT), aspartate transaminase (AST), base excess (BE), international normalized ratio of thrombin time (INR), diastolic arterial blood pressure, systolic arterial blood pressure (BPdia, BPsys) and activated partial thromboplastin time (aPTT)).

**Conclusions:**

The identified clinical phenotypes reflected varying degrees of sepsis severity and were mirrored in the plasma proteome. Proteomic profiling offered novel insights into the molecular mechanisms underlying sepsis and enabled a deeper characterization of the identified phenotypes.

**Supplementary Information:**

The online version contains supplementary material available at 10.1007/s15010-025-02628-3.

## Background

In recent decades, the major improvements in sepsis therapy were almost exclusively based on the quick application of an adequate infectiological treatment, and hemodynamic stabilization through fluid resuscitation and application of vasopressors [1]. However, with high mortality rates [2], sepsis remains a major burden in global healthcare. Apart from infectious disease and symptomatic treatment, specific therapeutic approaches directly addressing the molecular alterations in sepsis, have consistently failed or shown contradictory results [3, 4]. One possible explanation for these findings is seen in the fact, that clinical sepsis trials enrolled heterogeneous patient populations [5], leading to non-comparable sepsis manifestations. The identification of clinically relevant sepsis phenotypes might therefore be essential for the future development and predictive enrichment for specific sepsis therapies [6].

These days, a large amount of routine intensive care data is widely available, so that unsupervised machine learning (ML) methods appear to be well suited for identifying clinical sepsis phenotypes [7]. However, clinical phenotyping relies on routine data which serve as surrogate parameters rather than capturing the true underlying biomolecular alterations in sepsis. As a result, the identified subtypes offer limited potential for informing novel, personalized therapeutic strategies. Despite the promise of unsupervised clustering to reveal subgroups of sepsis, many existing approaches yield cohort-specific phenotypes. Differences in patient populations, measurement methods, and the variables chosen for analysis make it difficult to compare or replicate these clusters across different studies, resulting in a proliferation of clusters that often cannot be easily harmonized. To date, there is only limited information on the biomolecular characteristics that are associated with clinical phenotypes, thus limiting the opportunities for development of therapeutic strategies [6]. Moreover, since the course of sepsis is a highly dynamic process, temporal shifts in sepsis subtypes within individual patients may be a key factor in distinguishing critical from non-critical disease trajectories. Despite this potential, the temporal dimension of sepsis progression has so far rarely been studied and been largely neglected [8].

In this study, we aimed to (i) identify distinct sepsis phenotypes by unsupervised machine learning using routine clinical data, (ii) translate these subtypes to the molecular level to reveal biomolecular alterations relevant for personalized therapies by mass spectrometry-based plasma proteomics, (iii) analyze the trajectories of patients through these subtypes in accordance with their outcome, and (iv) develop and evaluate a supervised machine learning model that reliably detects these subtypes at the onset of sepsis treatment using a minimal set of universally accessible clinical variables, thereby enabling consistent and reliable phenotype assignments even across diverse cohorts and laying the groundwork for a future clinical decision support system.

## Methods

### Patient cohort

This work is based on the multicenter, prospective, observational SepsisDataNet.NRW and CovidDataNet.NRW studies (German clinical trial registry, no. DRKS00018871). Patients were eligible for inclusion when treated for sepsis meeting sepsis 3 criteria [9] within the previous 48 h at time of inclusion. In CovidDataNet.NRW patients were included when the inclusion criteria were complemented by an active SARS-CoV-2 infection. Suspected or proven infection was identified by the treating physician who also initiated an appropriate antibiotic therapy. Patient-related ICU data from electronic health records was collected and blood samples were obtained regularly. Therapy was not affected by study inclusion and was based on latest sepsis guidelines [1]. Parts of the cohort have already been analyzed in other contexts [10, 11].

### Cytokine measurements

The concentrations of 13 cytokines were quantified in serum samples using the LegendPlex Human Inflammation Panel 1 (Biolegend, San Diego) according to the manufacturer’s instructions. Briefly, the LegendPlex beads were incubated with serum samples, subsequently washed, incubated with detection antibodies, and washed again. Measurements were carried out using a flow cytometer (Canto II, BD Biosciences, CA) and concentration were interpolated from calibration curves. Values below the lower limit of quantification were considered to be 0 ng/mL, values higher than the upper limit of quantification were replaced with the respective upper limit of quantification.

### Clustering

Clinical variables on the day of sepsis diagnosis which had the following characteristics were selected for clustering: (1) routine assessment in the ICU, (2) less than 30% missing values (3) representation of the clinical severity of organ damage rather than an association with a treatment regime (Supplementary Tables 1 and 2). For variables with multiple observations per day data was aggregated by the median, minimum or maximum, using the most clinical informative value (e.g. minimum value for quick value and maximum value for creatinine). Missing values were imputed using data from the subsequent two days. If no corresponding values were available, the median of the respective variable was used for imputation. In addition to the clinical variables, the site of infection and the age of the patients were used for clustering. To adjust the weighting of different numerical values, the data was z-transformed before Principal Component Analysis (PCA) was carried out for dimension reduction with the aim to prevent highly correlated features disproportionately influencing the clustering process. Different sepsis phenotypes were identified using the k-means algorithm on the PCA results of the first 11 components (> 70% of variance). The number of clusters were identified using silhouette curves, finding three different clusters (Supplementary Figs. 1–3). For the time series, the data from days 4, 7, and 9 were transformed using the standardization parameters (mean and standard deviation) of day 1 to ensure consistent transformation across the timepoints. The resulting principal components of day one were used to project the data from the subsequent days into the same reduced feature space. To assign the data from the following days to the clusters, the Euclidean distance to each cluster center was calculated, and each data point was assigned to the cluster with the nearest cluster center. Calculations were done using Python (v.3.11.5) and the packages pandas (v.2.1.1) and scikit-learn (v.1.3.1).

### Plasma proteomics

A detailed description of plasma proteomics analyses can be found as supplementary material. Briefly, 1 µl plasma per sample was digested with trypsin according to the SP3 protocol as described before [12]. 455 samples from 301 patients and two time points were analyzed by LC-MS/MS in setups using either an Orbitrap Fusion Lumos or an Exploris 240 mass spectrometer (Thermo Scientific, Bremen, Germany). Data were acquired in data-independent acquisition mode and analyzed using DIA-NN (v.1.8.1) with a spectral-library that was generated with FragPipe (v.18) searching the UniProt/SwissProt data base restricted to *Homo sapiens* (v.2022_05). Sample batches were processed individually and subsequently normalized for batch effects as described before [12] (Supplementary Fig. 4). Differences in protein intensities between the clusters were tested for significance by ANOVA followed by Tukey’s post-hoc test. Proteins with a minimum of five observations per cluster were considered for testing. ANOVA p-values were corrected using the method of Benjamini-Hochberg. Proteins with an ANOVA p_FDR_ value and a post-hoc p-value ≤ 0.05 were considered significant. Functional annotation and enrichment analyses were carried out using the STRING web interface (string-db.org, v.12.0).

### Machine learning

For machine learning, routine ICU data with less than 30% missing values were used (Supplementary Tables 1 and 3). Data pre-processing, such as variable aggregation and imputation, was done using the same approach as in the clustering process. Before model development, highly correlated features were removed by calculating the Pearson correlation between features and excluding one of the two features with an absolute correlation greater than 0.7 (Supplementary Table 4). To select the most relevant features, a multiclass classifier based on a Random Forest model was employed. The data were split into training and test sets (70% training, 30% test), and a 100 times Monte Carlo Cross Validation (MCCV) [13] was performed to evaluate model metrics for different feature sets. The order of features for iterative training was determined based on an additional 100 times MCCV in the training dataset (70% training, 30% validation, Supplementary Fig. 5). In this process, the median ranks of feature importance were computed using SHAP values (Shapley Additive Explanations). Missing values were imputed using the median in each MCCV iteration. Cluster C was bootstrapped to 60% of the size of Cluster A in every training set of each MCCV to prevent a bias towards the over-represented cluster. To determine the optimal number of features, the averaged recall was plotted against the number of selected features. The point at which the recall growth rate markedly declined was identified using the *KneeLocator* function of the keed package in Python. Tune Hyperparameters can be found in Supplementary Table 5. Calculations were done using Python (v.3.10.12) and the packages pandas (v.2.2.2), numpy (v.1.26.4), keed (v.0.8.5), shap (v0.46.0) and scikit-learn (v.1.5.1).

## Results

A total of 384 patients were enrolled within 48 h of sepsis onset. Of these, 48 patients (12.5%) were included on the same day sepsis began, 265 patients (69.0%) were included on the following day, and 71 patients (18.5%) on the second day (still within 48 h). Hospital-acquired sepsis was identified in 70.1% of the cohort (*n* = 269). The most frequent comorbidities were arterial hypertension (68.0%, *n* = 261), cardiovascular diseases (40.9%, *n* = 157), diabetes mellitus (31.0%, *n* = 119), and obesity (28.4%, *n* = 109). A history of malignancy was present in 22.7% (*n* = 87), and 11.2% (*n* = 43) had undergone solid organ transplantation with subsequent immunosuppression. Chronic kidney disease requiring dialysis affected 4.9% (*n* = 19) of patients.

### Sepsis phenotypes

We identified three clusters representing different clinical sepsis phenotypes. These phenotypes showed a significant difference in the SOFA score, representing the *disease severity* of septic patients. Between the clusters, SOFA scores were gradually increasing from a median value of 7 (IQR 4–11) in cluster A (*n* = 202; 53%), over 10 (6–13) in cluster B (*n* = 156; 41%), to 15 (13–17) in cluster C (*n* = 26; 7%). The baseline characteristics with a focus on different organ systems affected by sepsis are presented in Table 1. No patients had a documented chronic liver disease in medical records at the time of inclusion. However, a systematic diagnostic work-up for liver comorbidities detecting also sub-clinical manifestations was not performed.

**Table 1:** Baseline characteristics of the study cohort, differentiated by sepsis phenotype

Characteristic	Total	Sepsis phenotype			*p*-value	post-hoc testing (*p* adjusted)		
		Cluster A	Cluster B	Cluster C		*p* (A vs. B)	*p* (A vs. C)	*p* (B vs. C)
Number of patients (%)	384 (100)	202 (53)	156 (41)	26 (7)	< 0.001			
Age [years]	66 (56–77)	61 (51–71)	70 (62–80)	72 (61–77)	< 0.001	< 0.001	0.025	0.565
Female sex (%)	151 (39)	81 (40)	58 (37)	12 (46)	0.651	-	-	-
Surgery-associated sepsis^1^ (%)	110 (39)	58 (35)	44 (44)	8 (50)	0.272	-	-	-
Focus of infection (%)					< 0.001	< 0.001	< 0.001	0.518
- intra-abdominal	90 (23)	29 (14)	51 (32)	10 (39)				
- bloodstream	21 (6)	5 (3)	12 (8)	4 (15)				
- urinary tract	21 (6)	6 (3)	15 (10)	0 (0)				
- pneumonia	126 (33)	78 (39)	43 (28)	5 (19)				
- CNS	9 (2)	9 (5)	0 (0)	0 (0)				
- COVID-19	75 (20)	58 (29)	16 (10)	1 (4)				
- other	42 (11)	17 (8)	19 (12)	6 (23)				
SOFA score	9 (5–12)	7 (4–11)	10 (6–13)	15 (13–17)	< 0.001	< 0.001	< 0.001	< 0.001
Septic shock (%)	87 (31)	29 (18)	43 (43)	15 (93.8)	< 0.001	< 0.001	< 0.001	0.001
30-days mortality (%)	151 (40)	58 (29)	69 (45)	24 (92)	< 0.001	0.011	< 0.001	< 0.001
- Survival time^2^ [d]	7 (2–14)	13 (6–19)	4 (2–12)	2 (0–3)	< 0.001	< 0.001	< 0.001	< 0.001
**Comorbidities (%)**								
Arterial hypertension	261 (68)	130 (64)	113 (72)	18 (70)	0.265	-	-	-
Cardiovascular disease	157 (41)	65 (32)	82 (52)	10 (39)	< 0.001	< 0.001	1.000	0.788
Diabetes	119 (31)	62 (31)	49 (31)	8 (31)	0.989	-	-	-
COPD	47 (12)	29 (14)	16 (10)	2 (8)	0.384	-	-	-
Obesity (BMI > 30 kg/m^2^)	109 (28)	55 (27)	48 (31)	6 (23)	0.628	-	-	-
Chronic Kidney Disease (CKD)	91 (24)	40 (20)	47 (30)	4 (15)	0.044	0.098	1.000	0.567
CKD with dialysis requirement	19 (5)	7 (4)	11 (7)	1 (4)	0.290	-	-	-
Malignant disease	87 (23)	43 (21)	40 (26)	4 (15)	0.408	-	-	-
Alcohol abuse	29 (8)	13 (6)	14 (9)	2 (8)	0.666	-	-	-
Nicotine abuse	67 (17)	37 (18)	28 (18)	2 (8)	0.396			
Solid organ transplantation	43 (11)	23 (11)	18 (12)	2 (8)	0.841	-	-	-
**Inflammation / Immunostatus**								
WBC [x10^3^/µL]	12 (8–18)	10 (7–16)	15 (10–20)	16 (9–20)	< 0.001	< 0.001	0.083	0.741
CRP [mg/dL]	14 (9–25)	13 (8–22)	19 (10–29)	14 (10–19)	0.008	0.011	0.027	0.336
Absolute lymphocytes count [x10^3^/µL]	1.0 (0.6–1.4)	1.0 (0.7–1.4)	0.9 (0.6–1.5)	0.8 (0.2–1.1)	0.488	-	-	-
Relative lymphocytes count [%]	9 (5–13)	9 (7–14)	6 (4–12)	9 (5–12)	0.022	0.019	0.917	0.917
Interleukin 6 [pg/mL]	260 (74–654)	136 (47–342)	399 (112–940)	2372 (447–7513)	< 0.001	< 0.001	< 0.001	0.010
Interleukin 8 [pg/mL]	78 (35–223)	50 (28–32)	101 (53–245)	736 (286–1127)	< 0.001	< 0.001	< 0.001	< 0.001
Interleukin 10 [pg/mL]	9 (3–27)	5 (2–11)	11 (4–27)	208 (119–386)	< 0.001	0.004	< 0.001	< 0.001
Interleukin 17 A [pg/mL]	0.6 (0.3-1.0)	0.5 (0.3–0.7)	0.6 (0.3–1.1)	0.9 (0.7–1.7)	< 0.001	0.129	< 0.001	0.028
Interleukin 18 [pg/mL]	347 (154–746)	210 (101–533)	437 (205–872)	1090 (510–3954)	< 0.001	< 0.001	< 0.001	0.007
CCL2 [pg/mL]	306 (152–582)	232 (130–440)	328 (183–588)	882 (372–1835)	< 0.001	0.023	< 0.001	0.062
Platelets count [x10^3^/µL]	195 (115–268)	204 (145–272)	185 (112–279)	76 (44–147)	< 0.001	0.058	< 0.001	< 0.001
Body temperature [°C]	37.6 (37.1–38.4)	37.6 (37.2–38.5)	37.6 (36.8–38.3)	36.9 (36.2–38.2)	0.021	0.143	0.027	0.138
**Renal**								
Creatinine [mg/dL]	1.2 (0.8–2.1)	1.0 (0.7–1.4)	1.6 (1.1–2.6)	2.1 (1.2–3.2)	< 0.001	< 0.001	< 0.001	0.256
Urea nitrogen [mg/dL]	25 (16–39)	21 (15–33)	31 (18–45)	32 (19–50)	< 0.001	< 0.001	0.041	0.965
Urine output [mL]	1340 (660–2200)	1690 (880–2440)	1000 (520–1940)	290 (60–500)	< 0.001	< 0.001	< 0.001	< 0.001
**Hepatic**								
Aspartate transaminase [U/L]	50 (25–121)	41 (24–68)	55 (29–160)	2432 (1091–5104)	< 0.001	0.002	< 0.001	< 0.001
Alanine transaminase [U/L]	30 (17–75)	26 (17–51)	33 (14–77)	1280 (652–2205)	< 0.001	0.132	< 0.001	< 0.001
Total bilirubin [mg/dL]	0.6 (0.4–1.2)	0.5 (0.3–0.9)	0.7 (0.5–1.5)	1.3 (0.9–1.9)	< 0.001	< 0.001	< 0.001	0.002
INR	1.3 (1.1–1.5)	1.2 (1.1–1.3)	1.4 (1.2–1.7)	3.3 (1.2–3.7)	< 0.001	< 0.001	< 0.001	< 0.001
**Cardiovascular / hemodynamic**								
MAP [mmHg]	80 (74–87)	85 (80–92)	75 (70–79)	79 (69–82)	< 0.001	< 0.001	< 0.001	0.118
Heart rate [/min]	84 (72–100)	81 (69–95)	86 (73–102)	103 (89–110)	< 0.001	0.020	< 0.001	0.007
Norepinephrine, maximum dose [mg/h]	0.4 (0.0-2.5)	0.0 (0.0-1.1)	0.6 (0.0–4.0)	9.0 (4.4–16.0)	< 0.001	< 0.001	< 0.001	< 0.001
Lactate [mmol/L]	2.4 (1.6–3.8)	2 (1.4–2.8)	3 (1.8–4.7)	15 (8–18)	< 0.001	< 0.001	< 0.001	< 0.001
NT-proBNP [pg/mL]	1728 (559–6133)	879 (357–2581)	4431 (1118–13904)	15,989 (6010–30604)	< 0.001	< 0.001	< 0.001	0.041
**Pulmonary**								
Mechanical ventilation (%)	229 (60)	121 (60)	87 (56)	21 (81)	0.055	-	-	-
Horovitz index [mmHg]	276 (179–379)	260 (163–367)	303 (208–398)	256 (186–316)	0.092	-	-	-
pH	7.3 (7.3–7.4)	7.4 (7.3–7.4)	7.3 (7.2–7.4)	7.2 (7.1–7.3)	< 0.001	< 0.001	< 0.001	< 0.001
pCO_2_ [mmHg]	48 (42–56)	49 (42–56)	46 (41–55)	51 (47–75)	0.125	-	-	-

Numeric values are represented as median and interquartile range (IQR), categorical values as absolute and relative frequencies. All values were obtained on the day of sepsis diagnosis. p-values were calculated by Chi-square test for categorical data and Kruskal-Wallis test for continuous data. The reported relative frequencies are based on the actual number of available data points for each variable. Post-hoc p values were corrected for multiple testing by Bonferroni method

^1^ Surgery prior sepsis (within the same treatment case). ^2^ Survival time for patients who died within 30 days

BMI: Body mass index. CKD: Chronic kidney disease. CNS: Central nervous system. COPD: Chronic obstructive pulmonary disease. COVID-19: Corona virus disease 2019. SOFA: Sequential organ failure assessment. WBC: White blood cells. CRP: C-reactive protein. CCL2: Chemokine (C-C motif) ligand 2. INR: International normalized ratio. MAP: Mean arterial pressure. NT-proBNP: N-terminal prohormone of brain natriuretic peptide. mmHg: Millimeter mercury

### Clinical representations of clusters

Least affected patients were found in cluster A, characterized by a 30-day mortality rate of 29% (*n* = 58/202) with a median survival time of 13 days (IQR 6–19). The *low* mortality in this group was clinically underpinned by just a moderate organ failure and moderate vasopressor support. Patients who were allocated to this cluster mostly had either a sepsis due to pneumonia or COVID-19. Moreover, cluster A included the youngest patients with a median age of 61 years (IQR 51–71).

Cluster B represented a sepsis phenotype between most and least affected patients. For patients allocated to cluster B on day 1 the median 30-day mortality rate was 45% (*n* = 69/156), with a median survival time of 4 days (IQR 2–12). The most common focus of infection in this cohort was located intra-abdominal. The main difference was the presence of just a moderate AKI. In comparison to cluster A, a higher rate of septic shock was evident.

**Fig. 1:**
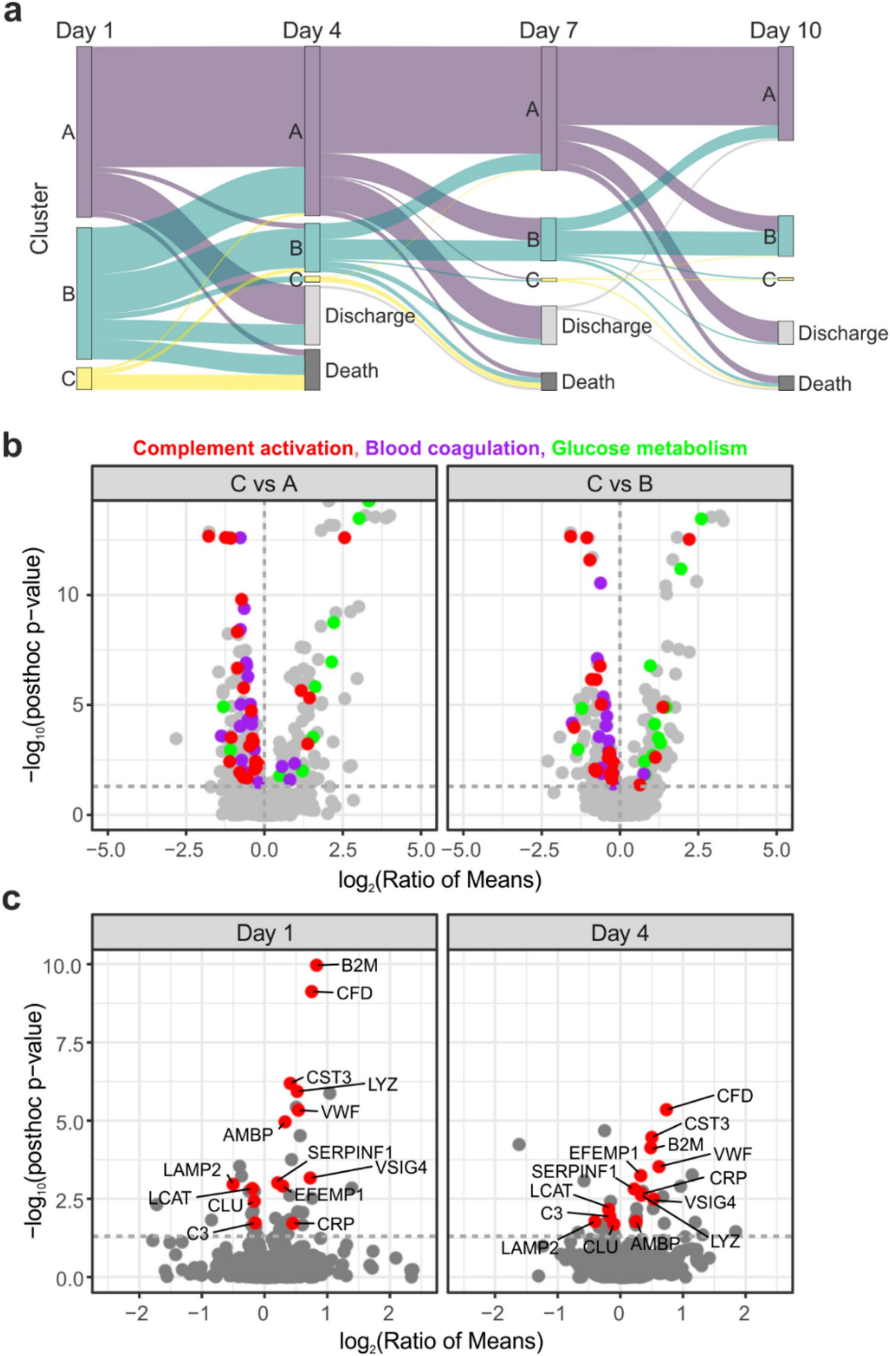
Time course analysis of clinical sepsis subtypes. **(a)** Sankey diagram illustrating the assignment of patients to clinical subtypes on days 1, 4, 7 and 10. Patients migrate between clusters or drop out of the analysis due to ICU discharge or fatal events. **(b)** Volcano plots illustrating the analysis of phenotypes A and B compared to C using ANOVA (Benjamini-Hochberg-corrected) followed by Tukey’s post-hoc test. Significantly differentially abundant proteins (p_FDR_ value ≤ 0.05, post-hoc p value ≤ 0.05) are annotated with the GO biological processes as indicated on the top. **(c)** Volcano plots showing the comparison of plasma proteome data between clusters A and B on day 1 and 4, respectively. Proteins that were found significant at both time points highlighted and labeled with gene names

The most affected patients were allocated to cluster C, which also showed up in a markedly high 30-day mortality rate of 92% (*n* = 24/26) with a median survival time of just 2 days (IQR 0–3). The cohort was suffering from multi-organ failure with a leading acute liver failure, lactate acidosis and accompanying high-grade acute kidney injury (AKI) with circulatory shock. The median age was 70 years (IQR 61–77). Accordingly, principal component analysis revealed the corresponding measures to have a major influence on the variation of the cohort (Supplementary Fig. 6, Supplementary Table 6). The longitudinal analysis revealed substantial dynamics between clusters, with Cluster B exhibiting the highest variability (Fig. 1). Notably, 39.7% of patients initially assigned to Cluster B transitioned to a different state within three days—either to the more favorable Cluster A (35.3%), the less favorable Cluster C (4.5%), or due to a fatal outcome (14.7%) (Supplementary Table 7). Cluster C showed a strong association with mortality with 97% of patients (all but one) migrating through it eventually dying within the 30-day period.

### Immunostatus

The immunological status of the sepsis phenotypes, showed the lowest relative lymphocyte count in cluster B with 6% (4–12), compared to clusters A and C with 9% each (IQR 7–14 and 5–12, respectively). Interleukins 6, 8, 10, 17 A, and 18 representing the inflammatory status of patients had the highest level in cluster C and the lowest in cluster A. This was also observed for chemokine (C-C motif) ligand 2 (CCL2) with levels of 232 pg/mL (130–440), 328 (183–588), and 882 (372–1835) for clusters A, B, and C, respectively.

### Plasma proteome characteristics of cluster C

**Fig. 2:**
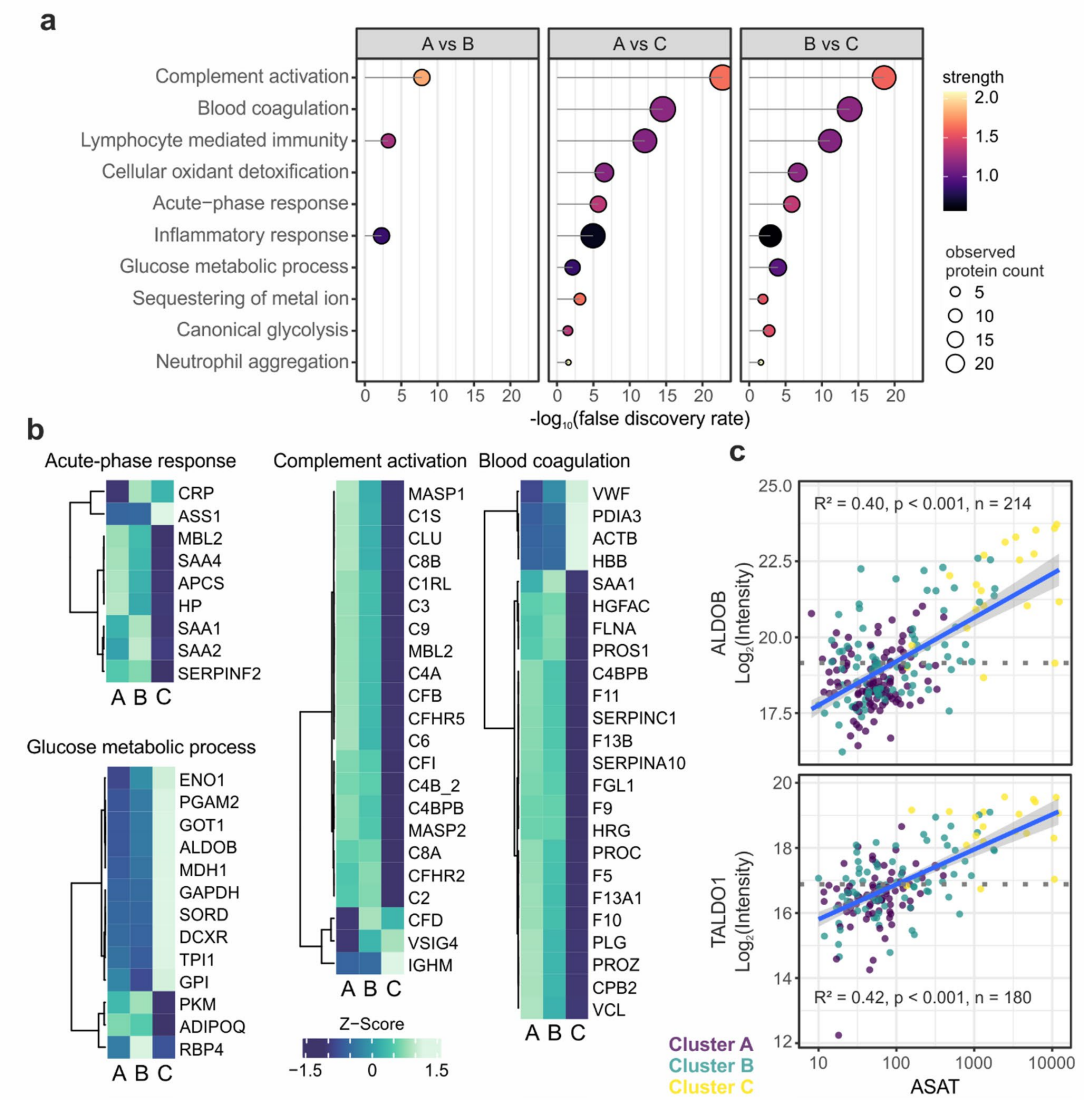
Proteomics characterization of clinical sepsis phenotypes at day 1. **(a)** Functional enrichment analysis based on GO biological processes. Analyses were performed separately for significantly differentially abundant proteins from the respective comparisons. Ten selected ontology terms are shown. **(b)** Heatmaps showing the mean intensities (z-scored) in the three phenotypes for all proteins, that were found statistically significant and are associated with the respective biological processes (Pearson correlation, complete linkage). **(c)** Scatter plots illustrating the linear regression analysis of protein intensities with aspartate aminotransaminase (ASAT) laboratory measurements. Blue line representing the linear fit with its confidence interval

We observed massive changes in the plasma proteome of cluster C in comparison to the other two clusters. Overall, we quantified 609 plasma proteins (Supplementary Fig. 7) of which at day 1 181 and 171 proteins were found significantly altered between clusters A and C and clusters B and C, respectively (Fig. 1b, Supplementary Table 8). The overlap between these proteins was 84% indicating specific proteome characteristics of cluster C. The differentially abundant proteins were associated with complement activation, blood coagulation and acute phase immune response (Figs. 1b and 2a). Strikingly, the related proteins were mainly lower abundant pointing towards an excessive consumption of complement and coagulation factors as well as acute phase proteins in the most affected patients (Fig. 2b). Of note, a decreasing intensity of the respective proteins was already apparent in cluster B, pointing towards a gradual decrease with increasing sepsis severity. Another major part of these protein alterations was associated with liver damage. We observed a striking enrichment of proteins related to glucose metabolism, probably released by liver cell death. The intensities of several proteins including fructose-bisphosphate aldolase B (ALDOB) and transaldolase (TALDO1) correlated with AST/ALT laboratory measurements (Fig. 2c). The association of these proteins with canonical glycolysis pointed towards a shift towards anaerobic metabolism in the liver. In addition, a limited synthesis capacity of the injured liver probably also contributed to the decreased abundance of complement, coagulation and acute phase proteins. At day 4, much less proteins were found to be differentially abundant in comparison to cluster C, which was a result of its small size with only five patients in the proteome analysis (57 proteins were altered between clusters A and C and 51 proteins between clusters B and C, Supplementary Table 8). Still, proteins related to blood coagulation and complement activation were prominently regulated and markers of liver injury were characteristic for cluster C. In addition, Myoglobin (MB) was substantially elevated in cluster C, indicating damage of muscle tissue [14], that was less prominent at day 1.

### Plasma proteome characteristics of clusters A and B

**Fig. 3:**
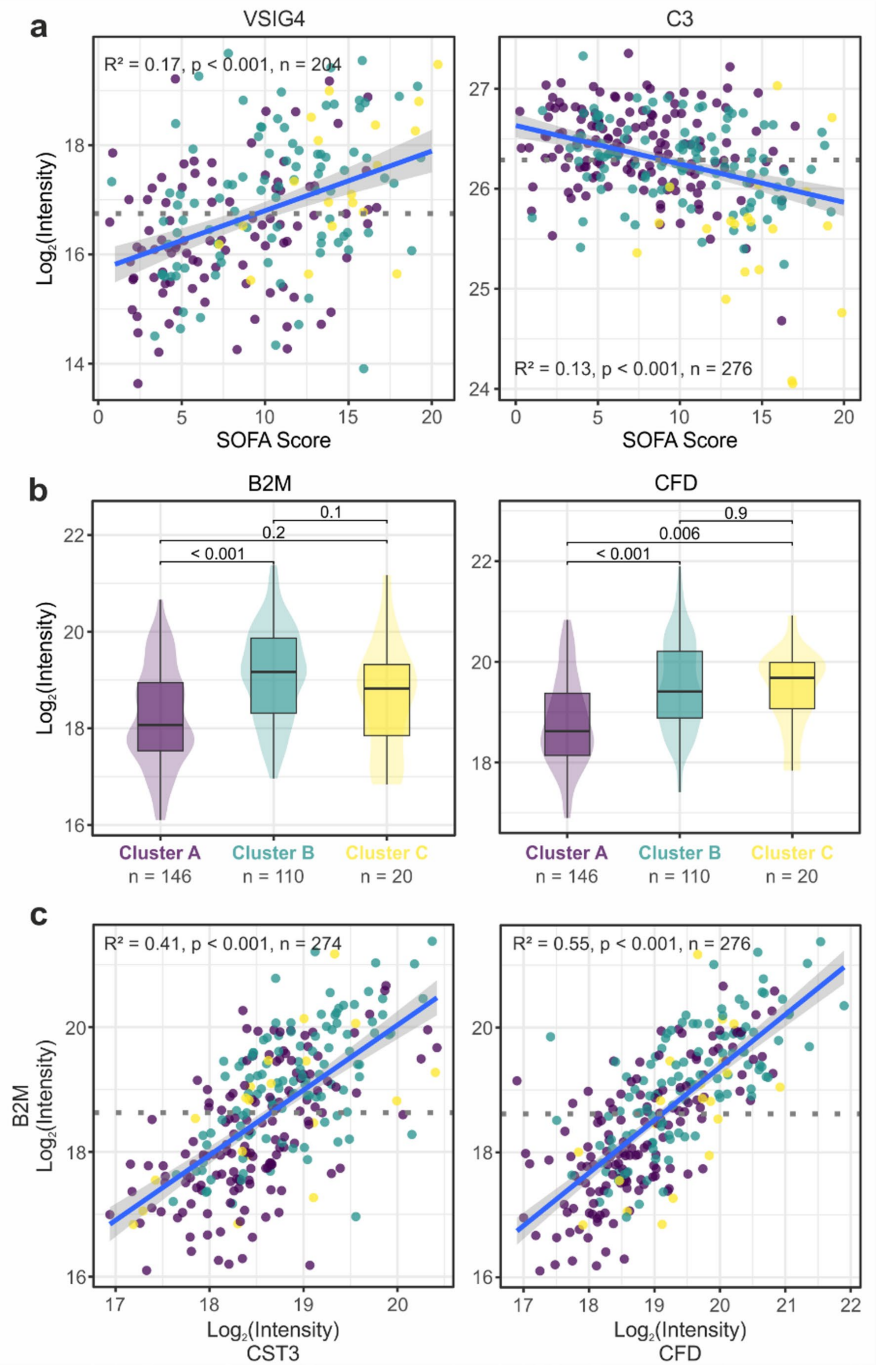
Comparison of clusters A and B at day 1. **(a)** Linear regression of V-set and immunoglobulin domain-containing protein 4 (VSIG4) and Complement factor 3 (C3), both significantly differentially abundant between cluster A and B, with the SOFA score. Colors of data points indicate the cluster the respective patients belong to. Blue line representing the linear fit with its confidence interval. **(b)** Boxplots illustrating the protein intensities of beta-2 microglobulin (B2M) and Complement factor D (CFD) in the three clusters. Boxes represent 25th and 75th percentiles, whiskers extend to the most extreme data points, median shown as a horizontal line, p-values from Tukey’s post-hoc test. **(c)** Linear regression analysis of B2M with Cystatin-C (CST3) and CFD

Clusters A and B showed less prominent differences with 40 differentially abundant proteins at day 1 and 29 proteins at day 4 (Supplementary Table 8, Fig. 1c). Several regulated proteins reflected the difference in disease severity between the clusters. The V-set and immunoglobulin domain-containing protein 4 (VSIG4), which was described to be secreted by activated peritoneal macrophages [15], for instance showed a significant correlation with the SOFA score and was consequently higher abundant in cluster B. Complement factor 3 (C3), on the other hand, was inversely correlated with the SOFA score (Fig. 3a). Other proteins reflected AKI as also indicated by increased serum creatinine levels in cluster B. The low-molecular-weight proteins complement factor D (CFD) and beta-2 microglobulin (B2M), which normally freely pass the glomerular filtration, were significantly increased in cluster B (Fig. 3b) and correlated with the established marker of renal function, Cystatin C (CST3), as well as with each other (Fig. 3c). Importantly, these proteins were found differentially abundant at both time points. Cluster A showed the mildest sepsis phenotype which was associated with the highest levels of complement and coagulation factors (Fig. 2b). This indicated the consumption of the corresponding proteins, which was already evident in cluster B and became excessive in cluster C.

### Patient classification by supervised machine learning

**Fig. 4:**
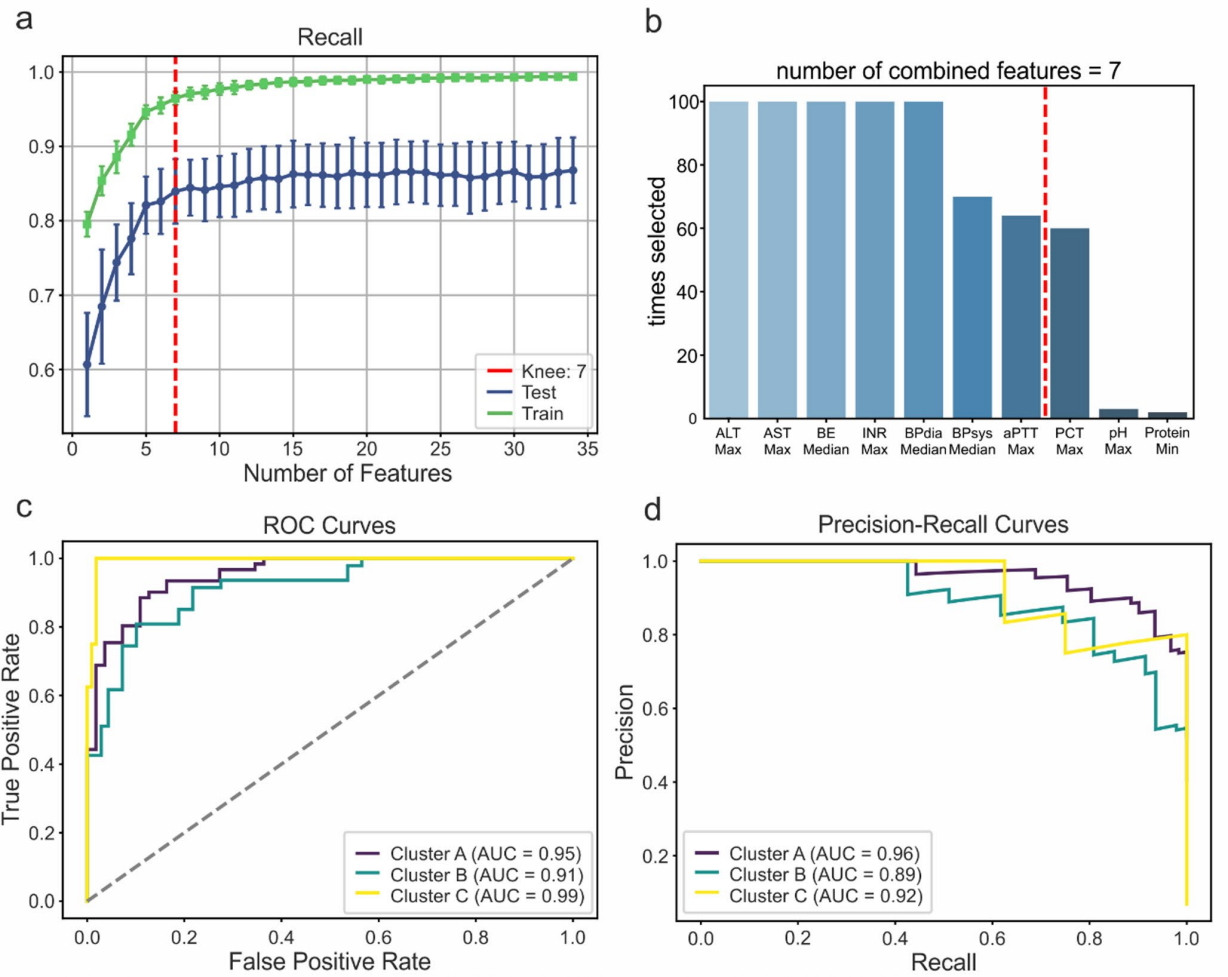
Model Performance of the Random Forest ML Classifier **(a)** Mean recall of the train and test data as a function of the number of features. The red dotted line indicates the computed knee point, where the slope decreases significantly at 7 features. **(b)** Bar chart showing the frequency at which a feature was included in a combination of seven features during MCCV. **(c)** Receiver Operating Characteristic (ROC) curves for all clusters, each compared to the rest, shown exemplarily from a single iteration during MCCV. **(d)** Precision-Recall curves for all clusters, each compared to the rest, shown exemplarily from a single iteration

For a possible future clinical application of our findings, it is essential to be able to identify the clusters in different cohorts, e.g. for assigning a sepsis patient currently undergoing treatment to a specific cluster. Since the cluster identification as described above relies heavily on the availability of the complete set of clinical data that we used for clustering, which is often not available in real-world scenarios, and to identify the most relevant features for discrimination between the three clinical phenotypes, we trained a random forest ML classifier and performed feature importance analysis. By iteratively adding features to the model and evaluating recall at each iteration, we identified the number of features beyond which only marginal improvement in recall was achieved (Fig. 4a). Over 100 iterations of Monte Carlo cross validation, the most frequently selected features in combinations with seven features were alanine transaminase (ALT), aspartate transaminase (AST), base excess (BE), international normalized ratio of thrombin time (INR), diastolic arterial blood pressure, systolic arterial blood pressure (BPdia, BPsys) and activated partial thromboplastin time (aPTT), all parameters which are widely available in clinical routine (Fig. 4b, Supplementary Table 9). Across all evaluated metrics, a discrepancy greater than 0.1 was observed between the training and test sets, indicating overfitting, likely due to an insufficient number of data points (Supplementary Table 10). Despite overfitting, an acceptable model performance can still be achieved for unseen data across all three classes with an AUROC of 0.95 ± 0.016 as well as a precision of 0.813 ± 0.050 and a recall of 0.839 ± 0.043 (Figs. 4c and d, Supplementary Table 10). These values should be interpreted with care, but they may serve as a proof-of-concept for future applications, demonstrating that the clusters can be predicted using only a few routinely collected features. Shapley feature importance analysis allows us to determine how individual features influence model predictions. In Cluster C, AST and ALT levels were elevated, while Clusters A and B differed in BE values, with higher BE levels favouring predictions for Cluster A (Supplementary Fig. 8). Furthermore, this approach provides insights into how specific features contribute to the prediction of a cluster for a given patient (Supplementary Fig. 9).

## Discussion

In the present study, we identified three clinical sepsis phenotypes and their temporal development in a prospective multicenter cohort using machine learning. The phenotypes represented different states of sepsis severity and differed in their clinical characteristics, the affected organ systems, and their immune status. Longitudinal analysis revealed dynamic characteristics of cluster B and a strong association with mortality of cluster C. We applied plasma proteomics to characterize the associated molecular alterations and found that the phenotypes translated to the plasma proteome. We could show how organ damage affected the composition of the plasma proteome and that components of the innate immune system depleted with increasing sepsis severity. For many of the related proteins we observed gradual changes along the clusters and an association with disease severity. Finally, we demonstrate that by using machine learning we are able to classify new patients by using just seven widely available clinical parameters while also interpreting the decision making.

Despite intensive research over the last decades, little progress has been made in sepsis therapy. High mortality and morbidity rates continue to bear witness to this [2]. Sepsis is based on an infection, but organ damage is an expression of pronounced dysregulation of the immune system and other metabolic processes [16]. Therefore, the most promising approach for future sepsis therapy is seen in the individualized and targeted treatment of metabolic and immunological alterations [17]. To advance precision medicine in sepsis, it is essential not only to identify clinical phenotypes but also to understand the biological mechanisms that underlie them. Previous work, has successfully stratified sepsis patients into clinical subtypes using machine learning on routine data [7, 18, 19]. However, these phenotypes were primarily based on surrogate markers (e.g., routine laboratory values) and lacked molecular characterization. On the molecular level, landmark studies have been performed based on transcriptome analyses of whole blood and immune cells as well as blood biomarker panels [20, 21, 22, 23]. The resulting subtypes were difficult to implement in clinics because the data sources lacked the widespread, quickly available features of clinical routine data. In contrast, our study bridges the critical gap between routine parameters that are available within shortest time and molecular insights by integrating high-throughput plasma proteomics, providing biological characterization to phenotypes defined by clinical data [24]. While direct proteomic phenotyping is currently not feasible at the bedside, we demonstrate that surrogate clinical phenotypes can offer meaningful biological insights if they are thoroughly characterized at the molecular level. This enables a biologically informed interpretation of clinical phenotypes and the early prediction of molecularly distinct disease courses using a minimal set of widely available clinical variables [25, 26, 27, 28], which lays the foundation for hypothesis-driven stratification in interventional trials.

To give a concrete example: In the plasma proteome of patients most severely affected by sepsis (cluster C), we found a deficiency in complement and coagulation factors to a previously unknown extent [29, 30], which was already apparent in cluster B, indicating a gradual decrease with increasing SOFA score. These findings contradicted previous observations [31], but differences in disease severity must be considered, given the overall higher SOFA scores in the de Nooijer et al. cohort, which presumably masked a gradual decrease in complement factors. While the coagulation system is well monitored by clinical measures, the complement system is not directly represented by routine data and its relevance in clinical diagnostics might be underestimated. This was underlined by the observation that complement factors were significantly reduced already in cluster B. Complement factor 3, a central hub of the complement cascade, showed a linear correlation with the SOFA score indicating a direct relation to sepsis severity rather than a secondary effect caused by impaired synthesis resulting from liver damage. Acute kidney injury, on the other hand, lead to the accumulation and elevated levels of low-molecular-weight plasma proteins, such as Complement factor D (CFD). CFD is the rate-limiting enzyme of the alternative pathway (AP) which is also responsible for signal amplification via the amplification loop [32]. Thus, the accumulation of CFD may further escalate the excessive activation of the complement system, which is also known to cause tissue damage and thrombo-inflammation [33]. Although the relevance of the complement is well established for COVID-19 [34] it is still largely overseen in polymicrobial sepsis and might represent a future target for therapeutic measures. Targeting CFD in cluster B would be an obvious strategy and inhibition of the AP amplification loop might benefit from limiting the destructive power of the complement cascade while preserving its basal activity and important functions such as leukocyte recruitment and activation [32].

Our findings suggest that identifying patients with specific biological profiles at the onset of sepsis may help to revive or refine targeted therapies. In this sense, longitudinal analysis of phenotypes is of great importance, as the time at which patients are assigned to phenotypes is influenced by many factors. Our data suggest that phenotypes in sepsis are stable over a significant period of time and show that this is also the case at the plasma proteome level. This observation is important if we hypothesize that clinical decision making should be supported by clinical phenotypes. On the other hand, we observed changes in phenotypes in a subset of patients that were associated with mortality when they migrated to cluster C, demonstrating that phenotypes could also help to monitor patients over the course of therapy. Importantly, we show that within these dynamics the molecular profile of the plasma proteome was consistent between phenotypes and the examined time points. Thus, our study not only provides a classification framework but also a biological rationale for future precision-guided clinical interventions [24]. Moreover, our machine learning models enable the simple allocation of patients to clinical phenotypes, offering a pragmatic approach to anticipate molecular profiles and guide phenotype-informed interventions.

### Limitations

A key strength of our work is the prospective enrolment of patients: inclusion occurred only when the treating physician confirmed a definite sepsis diagnosis, thereby minimizing the misclassification that can occur when sepsis is identified retrospectively in large secondary data sets such as MIMIC, where clinical adjudication is unavailable and over-diagnosis has been documented [35]. This advantage, however, is not unique to our study; several recent sepsis-phenotyping investigations have also relied on prospectively recruited cohorts—often with substantially larger sample sizes than ours. To our knowledge, ours is nevertheless the largest cohort in which machine-learning-derived clinical phenotypes have been further characterized by comprehensive plasma proteomics. The modest overall cohort size remains a limitation and may restrict the generalizability of our findings. Therefore, this study serves as a feasibility trial for a novel approach in characterizing sepsis. Hence, it is imperative that our results are validated in further cohorts.

Further limitations are that due to the unique composition of the plasma proteome and technical limitations of LC-MS/MS, it is currently extremely challenging to measure low abundant proteins, such as certain cytokines. Thus, our observations are limited to the top 600 plasma proteins that were covered by the present analysis.

Another key limitation is that enrolling patients within 48 h after onset of sepsis may introduce variability in the precision of their disease trajectory assessments, particularly for those recruited later in this time window. However, because the clustering criteria remain consistent across all time points, these effects likely have minimal impact on cluster assignments.

Additionally, because we did not perform a systematic liver diagnostic work-up at enrolment, undiagnosed or subclinical liver conditions could have been missed, potentially introducing residual confounding.

## Conclusions

In this study, we identified distinct clinical sepsis phenotypes that not only differed in disease severity, progression and outcomes, but also showed characteristic patterns in the plasma proteome, reflecting underlying metabolic and immunological alterations. While these findings emphasize the potential of combined clinical and molecular phenotyping to uncover biologically meaningful subgroups in sepsis, direct links between these proteomic differences and any specific therapeutic interventions remain to be established - particularly given that complement-targeted therapies have so far been unsuccessful. Further validation of our phenotypes in larger cohorts is urgently needed before translating these insights into clinical practice. Nevertheless, incorporating temporal dynamics and molecular data may help refine patient stratification, potentially guiding future phenotype-based studies and the development of more targeted interventional trials.

## Supplementary Information

Below is the link to the electronic supplementary material.

Supplementary Material 1

Supplementary Material 2

## Data Availability

The mass spectrometry proteomics data have been deposited to the ProteomeXchange Consortium via the PRIDE partner repository with the dataset identifier PXD058562.

## Abbreviations

AKI: Acute kidney injury
ALT: Alanine transaminase
AST: Aspartate transaminase
AP: Alternative pathway of complement activation
aPTT: Activated partial thromboplastin time
COVID-19: Coronavirus disease 2019
FDR: False discovery rate
ICU: Intensive care unit
INR: International normalized ratio
LC-MS/MS: Liquid chromatography-coupled tandem mass spectrometry
MCCV: Monte carlo cross validation
ML: Machine learning
PCA: Principal component analysis
SARS-CoV-2: Severe acute respiratory syndrome coronavirus type 2
SOFA: Sepsis-related organ failure assessment score
